# Exosomes and Exosomal Non-coding RNAs Are Novel Promises for the Mechanism-Based Diagnosis and Treatments of Atrial Fibrillation

**DOI:** 10.3389/fcvm.2021.782451

**Published:** 2021-12-01

**Authors:** Chaofeng Chen, Qingxing Chen, Kuan Cheng, Tian Zou, Yang Pang, Yunlong Ling, Ye Xu, Wenqing Zhu

**Affiliations:** Department of Cardiology, Zhongshan Hospital, Fudan University, Shanghai, China

**Keywords:** exosome, non-coding RNAs, atrial fibrillation, diagnosis, treatment

## Abstract

Atrial fibrillation (AF) is the most common arrhythmia worldwide and has a significant impact on human health and substantial costs. Currently, there is a lack of accurate biomarkers for the diagnosis and prognosis of AF. Moreover, the long-term efficacy of the catheter ablation in the AF is unsatisfactory. Therefore, it is necessary to explore new biomarkers and treatment strategies for the mechanism-based AF. Exosomes are nano-sized biovesicles released by nearly all types of cells. Since the AF would be linked to the changes of the atrial cells and their microenvironment, and the AF would strictly influence the exosomal non-coding RNAs (exo-ncRNAs) expression, which makes them as attractive diagnostic and prognostic biomarkers for the AF. Simultaneously, the exo-ncRNAs have been found to play an important role in the mechanisms of the AF and have potential therapeutic prospects. Although the role of the exo-ncRNAs in the AF is being actively investigated, the evidence is still limited. Furthermore, there is a lack of consensus regarding the most appropriate approach for exosome isolation and characterization. In this article, we reviewed the new methodologies available for exosomes biogenesis, isolation, and characterization, and then discussed the mechanism of the AF and various levels and types of exosomes relevant to the AF, with the special emphasis on the exo-ncRNAs in the diagnosis, prognosis, and treatment of the mechanism-based AF.

## Introduction

Atrial fibrillation (AF) is a most common type of cardiac arrhythmia and a global burden with significant morbidity, mortality, and socioeconomic problem (1, 2). The AF affects 1–1.5% of the population worldwide, the frequency of the condition is closely related to advancing age, and its prevalence is expected to more than double over the next 40 years (3, 4). Catheter ablation is an established treatment for AF, especially for paroxysmal AF (PAF). However, the success rate for the persistent AF (PsAF) is not ideal because the procedure is often accompanied by risks and other pathological complications. Moreover, there is a lack of effective upstream management for the AF (5–7).

Extracellular vesicles (EVs) include exosomes [diameter range (DR): 30–150 nm], microvesicles (DR: 50–1,000 nm) and apoptosomes (DR: 50–5,000 nm) (8). Exosomes are found in almost all body fluids (9–11). They normally contain lipids, proteins, and various RNAs, depending on the cells type and the cellular microenvironment (12, 13). Initially, exosomes were believed to be excretory vehicles to discard the metabolic waste but are now regarded as intercellular communicators that shuttle genetic information and proteins between cells (14, 15). The exosomal cargoes not only reflect the disease state, but also the physiological process of the receptor cells. Therefore, they can serve as unique biomarkers of developmental processes and prognostics/diagnostics of the disease states (16). Recently, the role of exosomes in cardiovascular diseases has been extensively studied, mainly in in the acute myocardial infarction (AMI), congestive heart failure (CHF), and coronary atherosclerotic disease (CAD), however, comprehensive elucidations on arrhythmia, especially on the AF are limited (17, 18). This review aimed to analyze the current knowledge regarding the exosomes' formation, isolation, biological functions, and advancements in the medical application, including potential diagnostic and therapeutic use in the AF.

## Exosome

### Exosome Biogenesis

**Figure 1:**
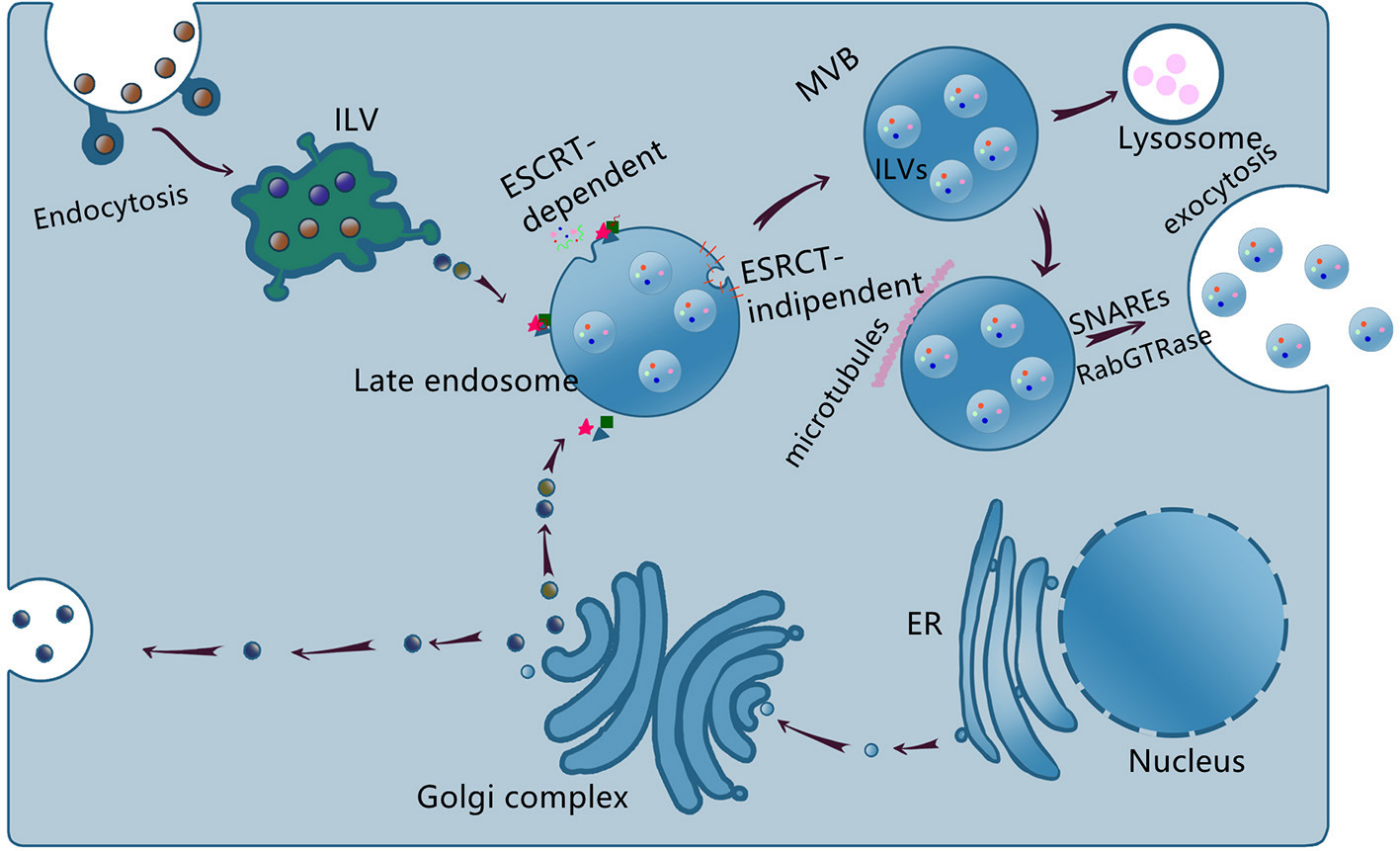
Schematic representation of exosome biogenesis, sorting, and release. The endosome membrane invaginates and sprouts to form intraluminal vesicle (ILV), the early endosome, and then matures to form multivesicular body (MVB) via ESCRT-dependent and ESCRT-independent, the late endosome. Some MVBs reach lysosome and the contents are degraded, others transported to the cell membrane to release exosomes via SNAREs and RabGTPase.

Exosome biogenesis and generation depend on the cell types or cellular microenvironments (19, 20). The exosome biogenesis is schematized in Figure 1.

Exosomes are formed by two invaginations of the plasma membrane. The first invagination generates early endosomes in the cytoplasm. The early endosomes mature into late endosomes, whose secondary invagination forms intraluminal vesicles (ILVs). Late endosomes and then finally form multivesicular bodies (MVBs). However, not all ILVs are released as exosomes and some of them would fuse with lysosomes and undergo degradation (21, 22). The exosome formation is tightly regulated by the endosomal sorting complex required for transport (ESCRT) and ESCRT-independent pathways. Exosome cargoes include proteins, lipids, and nucleic acids (23). In addition, nucleic acids especially non-coding RNAs (ncRNAs) serve as important cargoes and mediate cells communication (24–26). At present, the mechanisms underlying the cargoes sorting remain unclear.

The detailed mechanism of the MVBs intravesicular trafficking and fusion with the plasma membrane remains elusive. The known proteins involved are SNAREs, Rabs family (RAB27, RAB11, and RAB35), and Ras GTPase (27–30). The intravesicular trafficking may be mediated by the calcium-dependent Rabs family and the fusion process may be mediated by the SNAREs proteins (20, 25, 31). Exosomes are released into the extracellular space after fusion with the membrane. The release of the MVBs occurs in a calcium-dependent manner.

### Exosome Uptake

Possible consequences following the exosomes release include: (1) capture by the neighboring cells or re-absorption by their secretory cells; (2) remote relocation, recognition and fusion with the recipient cells membrane; (3) entry into the circulation and translocation to the other organs (32). However, the underlying mechanisms of the exosome uptake by the recipient cells remain debatable. As reported, there are three suggested mechanisms of uptake: internalization, direct fusion and receptor-ligand mediated uptake (33–40). Thereby, although the precise mechanism of exosome uptake is unclear, one fact remains obvious: the exosomes participate in cells communication through a complex intercellular exchange of biologically active molecules, modulating the function and behavior of the recipient cells. There is compelling evidence of this process occurring in a variety of diseases including cardiovascular diseases (11, 18, 20, 27, 32, 33, 41).

### Exosome Isolation

The isolation of pure exosomes is a critical step to understand their structures and physio-pathological roles in diseases. Nevertheless, there are no currently reliable protocols to isolate absolute pure exosomes. Although several methods have been used to isolate exosomes, each approach exhibits advantages and disadvantages (Table 1).

**Table 1:** Current available exosomes isolation techniques.

**Method**		**Pros**	**Cons**	**Procedure and application**
Ultracentrifugation		(1) The most commonly used method (2) Suitable for large sample capacity	(1) Time-consuming, costly instrumentation (2) Un-efficiently (3) Loss of large amount and damage of exosomes (4) Unsuitable for small amounts of samples or rare samples	It consists of a series of centrifugation cycles of different centrifugal force and duration to separate exosomes. Centrifugation is initially performed at a low speed, followed by ultracentrifugation at 100,000 to 120,000 × g. Finally, the isolated exosomes are resuspended in the appropriate medium. It is suitable for sample such as urine, ascites, and supernatant culture medium
Density gradient centrifugation	(1) Two step method	(1) High purity (2) Structure and function integrity	(1) Low yield and time-consuming (2) Unsuitable for large amounts of sample	The sample is usually layered onto the top of the density gradient medium and subjected to an extended round of ultracentrifugation. The vesicles travel through the gradient until they reach the point at which their density matches the one of the surrounding solution. The separated exosomes are then conveniently recovered by simple fraction collection. The process is suitable for scale analysis of exosomes
	(2) Single step method	(1) Integrity (2) Higher recovery yield	(1) Unsuitable for large amounts of sample	The conditioned medium containing exosomes was directly loaded on 30% sucrose gradient and centrifuged at 100,000 × g, 4°C for 90 min
	(3) Cushion combined with density gradient ultracentrifugation	(1) High purity (2) Preservation properties	(1) Time-consuming	Firstly concentrated by using 60% iodixanol cushion to maximize exosomes recovery. Then, the concentrated exosomes are separated through density gradient ultracentrifugation to remove non-exosomes contaminates
Size-based isolation methods [ultrafiltration, Size exclusion chromatography (SEC)]		(1) Rapid (2) No requiring centrifuge equipment	(1) Isolation of exosomes larger than the pore size of the matrix of the stationary phase used (2) Low yield and the purified sample is diluted (3) Significant hands-on time for column preparation, washing, and equilibration (4) Manual collection of fractions may introduce operator-dependent variability	It uses a stationary phase consisting of resin particles with known porous size. Similarly to density gradient centrifugation, SEC has been shown to allow reduction of contaminant proteins. The process is suitable for small scale analysis of exosomes
Immune-affinity purification		(1) High purity (2) Highly efficient (3) Maintaining exosomes specific morphology, biological activity, and molecular profiles	(1) Multiple steps in sample preparation, making the isolation prone to errors (2) PH value and salt concentration of the buffer might affect the biological activity of exosomes	Magnetic beads are widely used in this method for capturing anti-CD9, anti-CD63, and anti-CD81 antibodies and isolating exosomes
Polymer-based precipitation		(1) Easy, does not require any specialized equipment (2) High recovery rate (3) It is scalable for large sample sizes	(1) It contains lots of contaminating proteins (2) Polymer present in the sample may interfere with the downstream analyses	The sample is mixed with water excluding polymers, that tie up water molecules and force less soluble components out of solution. Generally, the biological fluid is incubated with a precipitation solution and, after incubation, the precipitate containing exosomes is isolated by low speed centrifugation. It is scalable for large sample sizes
Microfluidics based isolation Techniques	(1) Microfluidic based immunoaffinity capture approach (ExoChip)	(1) Highly efficient	(1) Not suitable for large volume, lack of method validation	Microfluidic devices exploit sample-bead interactions and subsequent separation of the beads. The sample is incubated with capture beads off-chip, and only downstream bead separation step takes place on-chip.
	(2) Microfluidics based membrane filtration approach	(1)Highly efficient and low cost	(1)Not suitable for large volume, lack of method validation	Devices use the micro-fluidics based membrane filtration approach isolating exosomes by their size.
				(1) the first such device is a nanoporous membrane with an adjustable pore size that inserted in a microfluidic chip; (2) a multiscale filtration device, which ciliated nanowire-on-micropillar structure that traps specifically sized liposomes (3) a type of microfluidic device based on pillar-array that can sort particles in a continuous flow through nano-deterministic lateral displacement.
Commercial kits		The Invitrogen isolation kit could isolate more and a broad size distribution of exosomes from the culture supernatant than the iZON gel-filtration chromatography kit, 101Bio PureExo kit, and affinity-based MagCapure kit. The quantity and quality of RNA isolated from exosomes showed no significant differences among these isolation kits. However, exosomes extracted using the Invitrogen kit appear to contain cytotoxic chemicals, which inhibit cell growth		

### Ultracentrifugation

Ultracentrifugation is the most commonly used method for exosome isolation. The process consists of a series of centrifugation cycles at different centrifugal forces and durations to separate exosomes from other components (42–45).

### Density Gradient Centrifugation

Density gradient centrifugation exploits differences in vesicle size and density through discontinuous density gradient layers with progressively decreased density from the bottom to the top (46–48).

### Cushion Combined With Density Gradient Ultracentrifugation

In this protocol, the exosomes are firstly concentrated using a 60% iodixanol cushion to recover a maximum number of exosomes with their property preserved. Then, the concentrated exosomes are separated through the density gradient ultracentrifugation to remove the non-exosomes contaminants (22).

### Size-Based Isolation Methods

Size-based isolation methods use filters (ultrafiltration) or chromatography columns and merely depend on size or weight. Size exclusion chromatography (SEC) is also a size-based separation technique that uses a stationary phase consisting of resin particles of known porous size to isolate exosomes (49–51).

### Immune-Affinity Purification of Exosomes

Exosome membranes contain large quantities of proteins. These proteins can be tagged by their specific corresponding antibodies to identify and isolate exosomes (45, 52–54).

### Polymer-Based Precipitation

Precipitation methods are easy and fast approaches for isolating exosomes, which use commercial kits. The exosomes are precipitated by altering their solubility in the solution (50, 55).

### Microfluidics-Based Isolation Techniques

Recently, microfluidics-based technologies have been introduced to identify and isolate exosomes. This technique exploits both physical and biochemical properties of exosomes, such as acoustic, electrophoretic, and electromagnetic characteristics (56, 57).

### Other Isolation Methods Using Commercial Kits

An increasing number of commercial kits are presently available for exosome isolation. Girijesh et al. analyzed these commercial kits regarding yield, purity, and downstream applications. They determined that the isolation kit by Invitrogen could isolate more exosomes from the culture supernatant than the IZON gel-filtration chromatography kit, 101-Bio PureExo kit, and affinity-based MagCapure kit. However, exosomes extracted using the Invitrogen kit contained cytotoxic chemicals, which may inhibit cell growth (58).

### Exosome Characterization

The characterization of exosomes has been a challenge due to their nano-scale size. So far, several techniques were employed for exosome characterization. The detail advantages, disadvantages and procedure are summarized in Table 2 (12, 59–71).

**Table 2:** Current exosomes characterization techniques.

**Method**	**Advantages**	**Disadvantages**	**Detectable size range**
TEM	High resolution, discriminate exosomes from other similar-size contaminants, immunostaining	Sample preparation may change the morphology of exosomes, potential damage by electron beam	>5 nm
NTA	Easy sample preparation, fast analysis, high resolution, vesicles are directly observed	Possible overlaying effect of larger vesicles, fail to distinguish exosomes from other nano-contaminants	50–1,000 nm
AFM	Minimal sample preparation without any destructive procedure	Scan speed, temperature and state of the tip may influent the analysis	>5 nm
DLS	High resolution	Fail to distinguish exosomes from other nano-contaminants	>5 nm
FACS	Able to identify specific EV subpopulations	Low detection sensitivity for EV	>300 nm
SEM	High-resolution imaging	Complex sample preparation Requires fixation and drying	>5 nm
TRPS	Information about surface charge of vesicles	Pores may be easily blocked by particles, generate a signal higher than the background noise of the system	>5 nm
Exoview platform	Small volume, low purification biases	Expensive instrumentation, time consuming	>5 nm
Flow cytometry	Fast analysis	Relate low resolution	Not available

*TEM, transmission electron microscopy; NTA, nanoparticle tracking analysis; AFM, atomic force microscopy; DLS, dynamic light scattering; FACS, fluorescence-activated cell sorting; SEM, scanning electron microscopy; TRPS, tunable resistive pulse sensing*.

## Mechanisms of AF

The mechanisms of the AF are complex and multi-factorial, and the pathophysiology includes three phases: initiation, maintenance, and progression (3, 72). Conceptually, these components link to the triggers and substrates. A trigger can act as an initiator, and the maintenance and progression generally require a substrate (73). Changes in substrate usually cause electrical and structural remodeling (74–76). In addition, a progression occurs over time from the trigger-driven disease, through the progress of atrial substrate, to the structural remodeling. These phases correspond to the clinical observation that about 5% of the patients with pAF progress to the persistent form each year, and 35–40% of PsAF patients may develop permanent AF within <1 year (77, 78).

### Triggers for AF

Three main mechanisms causing focal triggers are: enhanced atrial automaticity, early after-depolarization, and delayed after-depolarization (77, 79). In this regard, cellular calcium homeostasis may play an important role, which may cause heterogeneous electrophysiological properties, and then induce a vulnerable substrate formation (72, 74, 79, 80). These changes causing electrophysiological heterogeneity can result in initiation and sustenance of arrhythmia (72, 81).

### Substrate Changes for AF

Many theories about electrical remodeling have been proposed, and their common pathophysiological notion is reentry or micro-reentry (6). In myocardial AF, altered electrical property causes a shortening of the refractory period or slower conduction and thereby provides an anatomical substrate for reentry (5). Moreover, the structural changes such as dilatation and fibrosis of the atrium also affect the conduction and then maintain the reentry circuits (81, 82). Further, the calcium current is reduced by the inactivation and downregulation of the gene expression of calcium channels, which may lead to a shortening of the action potential (5, 83, 84).

### Atrial Fibrosis in AF

Extensive evidence shows that structural remodeling, particularly interstitial fibrosis, critically contributes to the substrate formation for the AF (6). Angiotensin-II mediates cardiac fibrosis in a variety of cardiac pathologies (85–87). The angiotensin II induces the TGF-β1 synthesis, which potently stimulates fibroblast activity. Moreover, the platelet-derived growth factor (PDGF) and connective tissue growth factor (CTGF) can also stimulate fibroblast proliferation and differentiation (74, 88–90).

### Atrial Apoptosis in AF

All cellular lineages undergo programmed cell death, but the fibrillating atria are more prone to apoptotic activation (91). It is likely that the apoptotic process begins relatively early in the AF and causes tissue remodeling (88, 92). Evidence from experimental models suggests that apoptosis, leukocyte infiltration, and increased cell death occur early and precede the arrhythmogenic structural remodeling (93).

### Immune Response in AF

The relationship between immune response and the AF is multiplex (94). Recently, several elucidations have shown that higher levels of inflammatory mediators and immune cells infiltration and are closely related to the AF (95). Inflammation could regulate calcium homeostasis and connexin expression, which in turn change the atrial substrates and cause AF initiation, and maintenance (96). The TNF not only could induce abnormal Ca^2+^ handling and arrhythmogenicity in pulmonary vein and cardiomyocytes, but also could activate the TGF-β signaling pathway in the myofibroblasts and increase the matrix metalloproteinase (MMP)-2 and MMP-9 secretion (97). The IL-2 can change the amplitude of electrically stimulated and caffeine-induced Ca^2+^ transients in myocytes. Inflammation also could alter the atrial conduction properties and increase the conduction heterogeneity by affecting the expression or distribution of the gap junction protein connexin (Cx) (Cx40 and Cx43), thereby inducing and maintaining AF (98). The leucocyte activation and increased levels of myeloperoxidase could increase the MMP-2 and MMP-9 activity, which then mediate atrial fibrosis and remodeling (99). Moreover, inflammatory mediators are associated with atrial electrical properties. The CD36 levels are positively correlated with the atrial voltage (100). Low levels of the HSP27 or CRP are associated with low atrial voltage (101).

### Atrial Myocardial Ischemia for AF

Acute myocardial infarction (AMI) is often accompanied by AF (102). The incidence of new-onset AF among AMI events varied from 4.5 to 10.9% in clinical settings (103). The mechanism of new AF in AMI is multi-factorial, among which acute atrial ischemia (AAI) caused by AMI plays an important role (104). AMI would cause electrical instability of ventricular cardiomyocytes, causing ventricular tachycardia or ventricular fibrillation (105). Similarly, AAI can also easily cause electrical conduction disorders in atrial cardiomyocytes, thereby increasing the susceptibility to atrial fibrillation (106). Therefore, increasing the blood supply after AAI may have a positive effect on preventing and reducing the occurrence of atrial fibrillation in these patients.

## Different Expression of Exosome in AF

In the AF, the cardiomyocytes and their microenvironment in the atria are in diverse pathological states. Because the biogenesis and secretion of exosomes significantly depend on the cellular conditions of the cardiomyocytes, the AF may cause changes in the exosomes profile and their cargoes in the atrial tissue and circulation (107).

Comparing the profile of circulating microparticles (MPs) between the AF patients and individuals with normal sinus rhythm (SR), Siwaponanan et al. found that the AF patients had significantly higher levels of cMPs (92). In addition, the EVs were measured in 836 patients with AF and in a cohort of control individuals in a study by Thulin et al. They showed that higher EVs were seen in anticoagulated patients with AF and a higher risk of stroke than the control population, possibly due to the high burden of AF (108). Moreover, Wang et al. found that the PsAF patients had a significantly increasing number of circulating microvesicles. Therefore, AF can cause different levels of circulating exosomes, especially PsAF (109).

Therefore, patients with AF have significant differentially expressed (DE)-exosomes, and the exosomes cargoes may be related to pro-inflammation, pro-fibrosis and apoptosis, which are important mechanisms of AF. Therefore, the exosomes may play a role in facilitating AF.

## Clinical and Biomedical Values of Exosome in AF

As stated previously, exosomes have been suggested as novel vehicles for intercellular communication in the cardiovascular system (71). Non-coding RNAs (ncRNAs) have emerged as important regulators of cardiac functions and diseases (110). So, the ncRNAs as important cargoes of exosomes, the exosomal ncRNAs (Exo-ncRNAs) should play an important role in the AF pathological process and can be used as diagnostic markers or in the treatment approach (111).

### NcRNAs in AF Progression

NcRNAs mainly include miRNAs, long non-coding RNAs (lncRNAs) and circular RNAs ect. MiRNAs are small ncRNAs of 22–24 nucleotides that are capable of regulating gene expression by interacting with the mRNA transcript 3'UTRs and promoting mRNA degradation and/or protein translation blockage (112). LncRNAs are a more diverse group of ncRNAs, providing transcriptional and post-transcriptional roles and subclassified according to their functional properties (113). CircRNAs are a closed continuous loop, function as sponges for miRNAs to regulate the expression of target genes and directly regulate transcription with RNA Pol II or protein coding (110, 114). We summarized current state-of-the-art knowledge on the functional of ncRNAs and their regulatory mechanisms in AF.

### miRNAs in AF

Many miRNAs are involved in cardiac remodeling, some of them regulate the ion channels, connexins or other proteins involved in the electrical remodeling, some regulate pro- or anti-fibrotic signaling cascades leading to the structural remodeling.

MiR-1 was down-regulated in the PsAF patients, accompanied by the up-regulation of *KCNJ2* and IK1 density, which was associated with the shortening of the action potential duration (APD) and enabled the reentry and AF maintenance (115, 116). MiR-26 was also down-regulated in the fibrillating atria, causing an up-regulation of transient receptor potential cation 3 (TRPC3) channels, which regulated the calcium influx, cell proliferation, extracellular signal-regulated kinase phosphorylation in the cardiac fibroblasts (117, 118). Recently, down-regulation of miR-29b and miR-106b-25 cluster (miR-25, miR-93, and miR-106b) was found in the AF patients atrial (119–121). MiR-30c and miR-133 down-regulation were accompanied by increased atrial fibrosis, and upregulation of their target gene *CTGF*, a pro-fibrotic mediator (122, 123). Besides, the MiR-133 was significantly down-regulated after the zinc finger homeobox 3 (ZFHX3) was knocked down, which increased the remodeling by targeted pro-fibrosis signaling (124). Additionally, up-/down-regulation of miR-133/miR-590 resulted in down-/ up-regulation of their target gene TGF-β1/TGF-β R II collagen expression (125). MiR-21 was up-regulated in the cardiac fibroblasts, which aggravated the pro-fibrotic ERK-MAP kinase signaling pathway (126–128). MiR-328 was also up-regulated in the AF patients' atrial tissue. The over-expression of miR-328 could lead to L-type calcium current reduction and APD shortening, increasing the AF vulnerability (129, 130). MiR-499 was elevated in fibrillating atrial tissue. A relationship was found between the miR-499 and *KCNN3*, which may have been involved in the AF pathophysiology (131). Moreover, the miR-499 mediated the AF by altering the mitochondrial fission and apoptosis signaling (132). MiR-208 can target the gene *GJA5* encoding the cardiac Cx40, and therefore mediate the pro-arrhythmogenic remodeling (133–136).

### Long Non-coding RNAs in AF

LncRNAs are involved in gene expression and cellular activity through a variety of mechanisms. Dysregulation of lncRNAs may be associated with cardiac diseases.

Based on competing endogenous RNAs' (ceRNAs) hypothesis, RP11-296O14.3 may participate in the AF pathological process (137). The lncRNA TCONS_00106987 was found increased in a rabbit AF model, which promoted the electrical remodeling by sponging miR-26 to regulate the *KCNJ2* (138). The lncRNA MIAT/TCONS_00202959 had an increase/decrease in fibrillating atrial tissues. The MIAT may target the miR-133a-3p to regulate the atrial fibrosis, and TCONS_00202959 may elongate the atrial effective refractory period (AERP) to decrease the AF inducibility (139, 140). Xu et al. (141) found that the lncRNA NONHSAT040387 and NONHSAT098586 were the most DE-lncRNAs in the AF patient blood samples. In another study, 19 DE-lncRNAs were identified from the AF patient monocytes, and the lncRNA HNRNPU-AS1 was the highest positive correlated one. Further, GO and KEGG analyses showed that these DE-lncRNAs were mainly involved in the metabolic, biosynthetic, RNA binding, NF-kappa B, and cytokine-cytokine receptor interaction signaling pathways (142). Additionally, the lncRNA GAS5 was found downregulated in the AF patients, and the change of the GAS5 occurred prior to the left atrial enlargement. Moreover, the GAS5 was negatively correlated to the ALK5, which could enhance the AF progression (143, 144). Besides, the lncRNA VDAC2P2, PVT1, NEAT1, PCAT-1, LICPAR, and NRON were increased in the AF patients, which were positively correlated with the collagen production and fibroblasts proliferation (145–151). However, the lncRNA LINC00472 and HOTAIR were downregulated. The LINC00472 could regulate the AF progression via modulating the miR-24/JP2/RyR2 signaling pathway, and HOTAIR could function as a ceRNAs in the Cx43 expression by sponging MiR-613 (151–154). In addition, NRON could alleviate atrial fibrosis through the suppression of M1 macrophages, promoting the M2 macrophage polarization. The lncRNA TCONS_00075467 could modulate the electrical remodeling by sponging miR-328 to regulate the *CACNA1C* expression (155). The lncRNA AK055347 may accelerate the AF pathogenesis by dysregulating the mitochondrial energy production via the regulation of Cyp450, ATP synthase, and MSS51 (156). Microarray and RNAs sequencing (RNA-seq) were employed in the lncRNAs analysis. The lncRNAs microarray of cardiac fibroblasts cells showed that the lncRNA AF159100, BC086588, and MRNR026574 were up-regulated while the MRAK134679, NR024118, and AX765700 were down-regulated (157). Another analysis showed that the lncRNA ENST00000559960/ uc004aef.3 was up-regulated/down-regulated in the AF patients' leukocytes (158). The RNA-seq analysis of lncRNAs in the AF canine cardiac fat pads showed that the TCONS_00032546 and TCONS_00026102 could shorten the AERP and increase the AF inducibility (159). The RNA-seq analysis in the AF patients showed that several DE-lncRNAs were involved in the signaling pathways associated with the PI3K/Akt, TGF-β, calcium, inflammation, oxidative stress, autophagy, apoptosis, and collagen synthesis (160, 161). Moreover, another RNA-seq data by Ke et al. identified that the lncRNA RP11-99E15.2 and RP3-523K23.2 participated in the AF pathogenesis via regulating the extracellular matrix binding and the transcription of the HSF2 (162).

### Circular RNAs in AF

Recently, studies showed a potential role of circRNAs in myocardial fibrosis and thus initiation and progression of the AF.

The circRNA-miRNA networks showed extensive interaction among DE-circRNAs and the AF-related miRNAs and mRNAs (163). The circRNAs microarray found 120 DE-circRNAs in the AF patients' monocytes. The circRNA_7571, circRNA_4648, circRNA_4631, and circRNA_2875 had the most binding nodes in the circRNA-miRNA networks and were closely interacted with the miRNAs (142). In addition, Gao et al. found that in the PsAF blood samples, circ_0004104 promoted cardiac fibrosis via the TGF-β pathway. Several other studies identified DE-circRNAs in the atrial tissues of AF patients (164). Zhang et al. identified 147 DE-circRNAs and GO and KEGG analyses indicated that many DE-circRNAs transcribed from the host genes were implicated in the regulation of sequence-specific DNA binding transcription factor activity (165). Zhang et al. (166) recognized 23 DE-circRNAs and circ_0000075 and_0082096 may participate in the AF pathogenesis via the TGF-β pathway. Another RNA-seq analysis found 296 DE-circRNAs and the circRNA-associated with the ceRNAs network may induce the AF through the cardiac muscle contraction alterations. Simultaneously, these DE-circRNAs may be involved in regulating the miR-208b and miR-21 expression (167). Another RNA-seq analysis in the patients with the PAF and PsAF found an increase of circRNAs from PAF transition to PsAF, accompanied by miRNAs down-regulation (168). According to an analysis of DE-circRNAs and ceRNAs network in the AF patients from the GEO database, 376 DE-circRNAs were identified, which were enriched in the cytokine-cytokine receptor interaction, and two ceRNAs pairs were identified (circRNA-100053- miR-455-5p-TRPV1 and circRNA-005843- miR-188-5p-SPON1) (169, 170).

### Exosomal-NcRNAs in AF

#### Exo-NcRNAs as Pathogenic Factors for AF

Many studies have found that exo-ncRNAs are related to the initiation and progression of Af. Myofibroblast-derived exo-miR-21-3p could reduce Cav1.2 expression, by regulating the AKAP/PKC signaling pathway, and then increase AF susceptibility (87, 171). Lu et al. found that exo-miR-328 could target the genes CACNA1C and CACNB1, which encode L-type calcium channels, and then lead to atrial remodeling (172). Shan et al. (125) showed that, in canines atrial fibroblasts, the decreased expression of exo-miR-133 and miR-590 were associated with atrial fibrosis, and then promoted AF. Epicardial fat (eFat) contains amounts of exsomes rich in pro-inflammatory and pro-fibrotic molecules, which can affect the neighboring atria, and induce the initiation and maintenance of AF (173–175). According to these researches, eFat tissues were collected from AF patients and were grown as organ cultures by Shaihov-Teper. eFat-EVs were isolated from the culture medium for further analysis. Moreover, to establish a causal association between eFat-EVs and vulnerability to AF, the study generated an *in vitro* AF model using induced pluripotent stem cell-derived cardiomyocytes (iCMs). The cultured explants from patients with AF secreted more EVs and harbored greater amounts of pro-inflammatory and pro-fibrotic cytokines, as well as pro-fibrotic miRNAs. Moreover, the eFat-EVs from patients with AF impacted the proliferation and migration of human mesenchymal stem cells (MSCs) and endothelial cells (ECs) and induced sustained reentry in iCMs (1). Some other studies also revealed that cardiomyocytes derived exo-miR-1, -miR-208a, -miR-21, -miR-223, -miR-26, -miR-29b, -miR-328, and -miR-499 could target pathways which involved in myocardial metabolism and remodeling (5, 172, 176). In short, these finding reveal the connection between exo-ncRNAs and the pathogenesis of AF, which may provide a promising alternative strategy to improving AF prevention and treatment.

#### Exo-NcRNAs as Diagnostic Biomarkers for the AF

Circulating miRNAs hold great promise as new diagnostic and prognostic biomarkers for cardiovascular diseases, but the specificity and sensitivity of the miRNAs could be affected by several factors. Due to the protection by the lipid bilayer membrane, circulating exo-miRNAs would provide stable miRNAs, and therefore, circulating exo-miRNAs may possess higher sensitivity and specificity to use as potential biomarkers for cardiovascular diseases (32). Nowadays, circulating exo-miRNAs as biomarkers were mainly used in the AMI, CHF, and CAD (exo-miR-150, -miR-320a, and -miR-208b ect.) (121, 177). Some studies have also found circulating exo-miRNAs could be used as diagnostic/prognostic biomarkers for AF. A study comparing circulating the exo-miRNAs between the patients with SR, PAF, and PsAF.

Wei et al. identified significant three DE-exo-miRNAs (miR-92b-3p, miR-1306-5p, and miRlet-7b-3p), and these miRNAs and target genes participated in AF pathogenesis, like as energy metabolism, lipid metabolism, inflammation, and enzyme activity (178). Wang et al. found that circulating exo-miRNAs: miR-483-5p, miR-142-5p, miR-223-3p were correlated with the AF and multivariate logistic analysis suggested that the miR-483-5p was independently in correlation with the AF (179). A study by Mun et al. also found that compared with patients with supraventricular tachycardia, the expression level of 45 circulating exo-miRNAs in patients with perAF was significantly increased (> 1.5 times). What's more, the DE circulating exo-miRNAs (miRNA-103a, miR-107, miR-320d, miR-486, and let-7b) were increased by more than 4.5 times in the PsAF. Moreover, these miRNAs and their target genes were involved in the atrial structure and function, oxidative stress, and fibrosis pathways (180). Further, Liu et al. isolated exosomes from pericardial fluid (PF), and found that the miR-382-3p, miR-450a-2-3p, and−3126-5p in the exosomes, and especially the miR-382-3p seemed pivotal in the AF progression (181). Therefore, circulating exo-miRNAs have the potential to serve as biomarkers in assessing the AF severity or prognosis, but more rigorous studies are necessary to confirm the supposition (Table 3).

**Table 3:** Exo-ncRNAs as potential diagnostic biomarkers and therapeutics approaches in pathogenic mechanism of AF.

**Exo-ncRNAs**	**Origination**	**Effect**	**Mechanisms**	**References**
Exo-miR-92b-3p/Exo-miR-1306-5p/Exo-miRlet-7b-3p	Plasma	Diagnostic	These miRNAs and target genes were involved in the process of AF through affecting biological processes such as energy metabolism, lipid metabolism, inflammation, and enzyme activity	(178)
Exo-miR-483-5p/Exo-miR-142-5p/Exo-miR-223-3p	Plasma	Diagnostic	Some of the pathways are related with myocardial remodeling (PI3K-Akt signaling pathway, adrenergic signaling in cardiomyocytes, focal adhesion, Wnt signaling pathway, calcium signaling pathway) and oxidative stress (MAPK signaling pathway, oxytocin signaling pathway)	(198)
Exo-miRNA-103a/Exo-miR-107/Exo-miR-320d/Exo-miR-486/Exo-miR-let-7b	Serum	Diagnostic	These miRNAs were involved in atrial function and structure (e.g., gap junction, adherens junction, adrenergic signaling), oxidative stress (e.g., MAPK, AMPK), fibrosis (e.g.,Wnt, hypoxia inducible factor-1), and other pathways	(180)
Exo-miR-382-3p/Exo-miR-450a-2-3p/Exo-miR-3126-5p	Pericardial fluid	Diagnostic	Implicated in cardiac fibrosis-related pathways, including the hypoxia-inducible factor-1 (HIF1), mitogen activated protein kinase (MAPK), and adrenergic and insulin pathways	(181)
Exo-Let-7c	MSCs	Treatment	Anti fibrosis, regulating the *TGF-β/Smad*	(183)
Exo-miR-17	CPCs	Treatment	Anti fibrosis, inhibit the TGF-β-induced fibrosis under oxidative stress	(184)
Exo-miR-19a	MSCs	Treatment	1) Anti-apoptosis, inhibit oxidative stress-induced apoptosis by targeting three prime untranslated regions in cylindromatosis, subsequently achieving the protective effect. 2) Anti-inflammation, decrease the expression of the inflammatory cytokines, moreover, pro-inflammatory/anti-inflammatory factors were down-regulated/up-regulated. 3) Anti fibrosis, downregulates the expression of the target proteins in CMs, *PTEN*, and *Bcl-2*-like protein, and activates the *Akt* and *ERK* signaling pathways	(71, 200)
Exo-miR-21	CPCs/ iPSCs/MSCs	Treatment	1) Anti-apoptosis, ameliorate the CMs apoptosis, which may relate to the inhibition of *caspase 3/7* mediated apoptosis by the *miR-21/PDCD4* signal axis. 2) Angiogenesis, induce angiogenesis and improve the cardiac cells' survival via inhibiting the *PTEN/Akt* pathway	(196, 197, 209)
Exo-miR-22	BMMSCs	Treatment	Anti fibrosis and anti-apoptosis, target the *Mecp2* to alleviate fibrosis and inhibit apoptosis	(185)
Exo-miR-24-3p	MSCs	Treatment	Anti-apoptosis, decrease apoptosis and promote the CMs proliferation	(201)
Exo-miR-25-3p	BMMSCs	Treatment	1) Anti-inflammation, inhibit the inflammatory cytokines expression. 2) Anti-apoptosis, inhibit apoptosis by *Ezh2/Socs3*	(22)
Exo-miR-26a	Muscle	Treatment	Anti fibrosis, blunt the *FOXO1* activation and inhibit cardiac fibrosis	(195)
Exo-miR-125b	BMMSCs	Treatment	1) Anti-apoptosis and 2) anti-inflammation, had the ability of anti-apoptosis and inhibit the inflammatory cytokines expression	(204)
Exo-miR-126	CD133^+^ cells/ CFs/ADSCs	Treatment	1) Anti fibrosis, reduce *VCAM, SPRED-1*, and *MCP1*, and subsequently decrease the interstitial fibrosis. 2) Anti fibrosis, inhibit fibrosis by targeting the f *TGF-β* and *collagen I*. Anti-apoptosis, reduce apoptosis in neonatal rats cardiomyocytes and improve cell survival by targeting *ERRFI1*. 3) Angiogenesis, promote the generation of microvascular cells and the migration of endothelial progenitor cells, through enhancing the *VEGF* pathway via the suppression of angiogenesis inhibitors *SPRED1* and *PI3KR2*	(190, 192, 211)
Exo-miR-132	CDCs	Treatment	1) Angiogenesis, inducing capillary-like tube formation and enhancing the migration and proliferation of HUVEC, through suppressing the expression of the *Efna3* and *RASA1*	(221)
Exo-miR-133a	NA	Treatment	1) Anti-apoptosis, inhibits apoptosis in myocardial ischemic postconditioning, prevents the expression of *TAGLN2* and *caspase-9*, and upregulates the expression of antiapoptotic protein *Bcl-2*	(201)
Exo-miR-144	MSCs	Treatment	Anti-apoptosis, target the *PTEN/AKT* pathway, and thus improve the apoptosis of the CMs	(202)
Exo-miR-146a	ADSCs/DCs/ CDCs	Treatment	1) Anti fibrosis, down regulating the gene *EGR1*. 2) Anti-inflammation, regulated the inflammatory response by inhibiting the *IRAK-1*. 3) Anti-apoptosis, targeting of *Irak-1* and *Traf6*, both involved in the toll-like receptor (TLR) signaling pathway	(182, 210, 215, 231)
Exo-miR-155	Macrophage/ECs	Treatment	1) Anti fibrosis, decrease fibroblast proliferation by inhibiting the *SOS-1*. 2) Anti-inflammation, can polarize macrophages to M2 cells, inhibit inflammatory reactions (KLF2/miR-155)	(191, 232)
Exo-miR-155-5p	Serum	Treatment	1) Anti fibrosis, enhances the *S1PR1* and inhibits the *SOCS1/STAT3* signaling pathway, thereby reducing the 2) Anti-inflammation, reduce the *IL-6* and *IL-17* in the valve tissue and serum	(214)
Exo-miR-181a	MSCs	Treatment	Anti-inflammation, create an anti-inflammatory environment and increase the Tregs polarization	(213)
Exo-miR-185	BMMSCs	Treatment	Anti-apoptosis and anti-inflammation, had the ability of anti-apoptosis targeting *Socs2*	(207)
Exo-miR-210	CPCs/MSCs	Treatment	1) Anti fibrosis, inhibit the TGF-β-induced fibrosis under oxidative stress. 2) Anti-apoptosis, downregulated its known targets, ephrin A3 and PTP1b, inhibiting apoptosis in cardiomyocytic cells. 3) Angiogenesis, inducing capillary-like tube formation and enhancing the migration through suppressing the expression of the *Efna3* and *RASA1*	(184, 205, 219, 220)
Exo-miR-221	MSCs	Treatment	Anti-apoptotic by inhibiting the *P53* and *Bcl-2b* and reducing the methylation of *CpG* binding protein-2	(206)
Exo-miR-223	BMMSCs	Treatment	Anti-inflammation, induce the expression of *ICAM-1* to inhibit the inflammatory reaction	(218)
Exo-miR-320	CMs	Treatment	Anti fibrosis, negatively affect the proliferation and migration of ECs	(188)
Exo-miR-320d	ADSCs	Treatment	Anti-apoptosis, negatively regulated *STAT3* expression, indirectly inhibited CMs apoptosis in AF, and increased survival, providing new insights into treatment strategies of AF	(17)
Exo-miR-423-3p	CFs	Treatment	Anti-apoptosis, improve the viability of the H2C9 and reduce apoptosis by targeting the *RAP2C*	(212, 213)
Exo-miR-290/Exo-miR-294/Exo-miR-295	ESCs	Treatment	Anti fibrosis, anti-apoptosis and angiogenesis, increases neovascularization improves cardiomyocyte survival and reduces fibrosis. Enhances cardiac progenitor cell survival and proliferation, as well as cardiac commitment	(186)
Exo-miR-378/Exo-miR-29a/Exo-miR-29b/Exo-miR-455	CMs	Treatment	Anti fibrosis, reducing the collagen and *MMP9* via inhibiting the *MAPK* and Smad pathways	(187)
Exo-miR-425/Exo-miR-744	Serum	Treatment	Anti fibrosis, inhibit fibrosis by targeting the f *TGF-β* and *collagen I*	(193, 194)
Exo-miR-181b/Exo-miR-182	CDCs/MSCs	Treatment	Anti-inflammation, reduce *PKCδ* transcription. Promoted the polarization of M2 macrophages and thereby alleviated the inflammatory response	(216, 217)
Exo-miR-150-5p/Exo-miR-142-3p/Exo-Let-7d	Tregs	Treatment	Anti-inflammation, reduce the immune reactions, and suppress the Th1 proliferation and secretion of the pro-inflammatory cytokines	(209, 223)
Exo-lncRNA Mhrt	ND	Treatment	Anti fibrosis, inhibit cardiac fibrosis and cardiac myocyte hypertrophy	(199)

*CMs, cardiomyocytes; ECs, Endothelial cells; CFs, Cardiac fibroblasts; CDCs, cardiosphere derived cells; MSCs, cardiac progenitor cells; ESCs, Embryonic stem cells; ADSCs, adipose-derived stem cells; BMMSCs, bone marrow derived cardiac progenitor cells; CPCs, cardiac progenitor cells; iPSCs, induced pluripotent stem cells. ND, Not Determined*.

#### Exo-NcRNAs as Potential Therapeutics Approaches in Pathogenic Mechanism of AF

There has been no research on the application of exosomes to the treatment of AF patients. Even in terms of animal experimental studies, direct data to prove the treatment of atrial fibrillation by exosomes-NCRNA is very limited. However, as mentioned previously, the mechanisms of the AF are closely linked to fibrosis, remodeling, inflammation, and apoptosis. In addition, acute atrial ischemia is always accompanied by AF. Therefore, the intervention on these mechanisms may provide a promising alternative new directions for AF treatment. Growing evidence suggests the role of exo-ncRNAs on these mechanisms, and therefore, the exo-ncRNAs may be used as the potential therapeutic tool for AF (18) (Table 3).

#### Anti-fibrosis

Adipose-derived stem cells (ADSCs)-exo-miR-146 could inhibit myocardial fibrosis by down-regulating the gene *EGR1* (182). The exo-Let-7c originating from the MSCs exhibits antifibrotic property, through regulating the TGF-β/Smad (183). The exo-miR-17 and miR-210 derived from the cardiac progenitor cells (CPCs) could inhibit the TGF-β-induced fibrosis under oxidative stress (184). Bone marrow-derived MSCs (BMMSCs)-exo-miR-22 could target the *Mecp2* to alleviate fibrosis (185). Moreover, exosomes enriched with the miR-290, miR-294, and miR-295 derived from the embryonic stem cells (ESCs) could significantly ameliorate fibrosis (186). Cardiomyocytes-exo-miR-378, miR-29a, miR-29b, and miR-455 could exert an anti-fibrotic effect by reducing the collagen and MMP9 via inhibiting the MAPK and Smad pathways (187). Moreover, the exo-miR-320 derived from diabetic cardiomyocytes could negatively affect the proliferation and migration of ECs (188, 189). Furthermore, CD133^+^-exo-miR-126 could reduce VCAM, SPRED-1, and MCP1, and subsequently decrease the interstitial fibrosis (190). Activated macrophage-exo-miR-155 has been shown to decrease fibroblast proliferation by inhibiting the SOS-1 (191). The miR-126, miR-425, and miR-744 enriched exosomes could inhibit fibrosis by targeting the f TGF-β and collagen I (192–194). Further, exo-miR-26a could blunt the FOXO1 activation and inhibit cardiac fibrosis (195). However, several exo-miRNAs have controversial properties. The exo-miR-21 and miR-181b could reduce or accelerate cardiac fibrosis under different conditions (196, 197). In RHD, the exo-miR-155-5p could reduce valvular fibrosis by inhibiting the SOCS1/ STAT3 pathway (25, 198). Moreover, lncRNA Mhrt was shown to inhibit cardiac fibrosis and cardiac myocyte hypertrophy (199).

Aforementioned, atrial fibrosis plays an important in atrial remodeling. A variety of exo-ncRNAs, especially derived from stem cells, can inhibit and improve myocardial fibrosis through a variety of pathways. Therefore, we believe that the treatment based-on these exo-ncRNAs may be an important strategy to prevent and treat AF by inhibiting fibrosis.

#### Anti-apoptosis

The exo-miR-320d from the ADSCs negatively regulated STAT3 expression, indirectly inhibited cardiomyocytes apoptosis in AF, and increased survival, providing new insights into treatment strategies of AF (17). The MSCs-exo-miR-19a could inhibit oxidative stress-induced apoptosis by targeting three prime untranslated regions in cylindromatosis (CYLD), subsequently achieving the protective effect (11, 200). Another exo-miRNA derived from the MSCs (exo-miR-24-3p) was also found to decrease apoptosis and promote the cardiomyocytes (CMs) proliferation (201). Under hypoxia, the MSCs-exo-miR-144 could target the PTEN/AKT pathway, and thus improve the apoptosis of the CMs (3, 202). Moreover, the exo-miR-210 and exo-miR-133a could inhibit apoptosis under hypoxia, by preventing transgelin 2 (TAGLN2) and caspase-9, and up-regulating the anti-apoptotic protein Bcl-2b. Simultaneously, it improved the ability to resist oxidative stress and supported the stem cells' survival (203, 204). Exosome-derived miR-210 downregulated its known targets, ephrin A3 and PTP1b, inhibiting apoptosis in cardiomyocytic cells (205). The BMMSCs-exo-miR-22 to reduce the methylation of CpG binding protein-2 and reduce cardiomyocyte apoptosis (186), and BMMSCs-exo-miR-221 could mediate the anti-apoptotic effect by inhibiting the P53 and Bcl-2b (206). Moreover, the BMMSCs-exo-miR-185 and exo-miR-125b had the ability of anti-apoptosis (207, 208). The exo-miR-21 originating from the CPCs and iPSCs was reported to ameliorate the CMs apoptosis, which may relate to the inhibition of caspase 3/7 mediated apoptosis by the miR-21/PDCD4 signal axis (183, 196, 209). The CDCs-exo-miR-146a could reduce scar formation after myocardial infarction in rats, inhibit cardiomyocyte apoptosis, and improve heart function (210). In addition, Wang et al. showed that the exo-miR-126 could reduce apoptosis in neonatal rats cardiomyocytes and improve cell survival (211). Cardiac fibroblasts-exo-miR-423-3p was also found to improve the viability of the H2C9 and reduce apoptosis by targeting the *RAP2C* (212).

Cardiomyocytes apoptosis can occur earlier than atrial remodeling. AF can also aggravate the apoptosis. Cardiomyocytes apoptosis and AF are a mutually deteriorating process. Early intervention for apoptosis may prevent and inhibit the initiation and progression of AF. Previous studies showed that exo-ncRNAs have important significance in improving apoptosis. Therefore, we believe that exo-ncRNAs with anti-apoptotic functions may have potential prospects in the treatment of AF.

#### Anti-inflammation

The MSCs-exo-miR-19a could decrease the expression of the inflammatory cytokines. In addition, the pro-inflammatory/anti-inflammatory factors were down-regulated/up-regulated by the treatment with the exo-miR-19a (71). The MSCs-exo-miR-181a could create an anti-inflammatory environment and increase the Tregs polarization (213). Moreover, the exosomes derived from Tregs could transfer the miR-150-5p, miR-142-3p, and Let-7d to dendritic cells (DCs) and T-helper 1 (Th1), reduce the immune reactions, and suppress the Th1 proliferation and secretion of the pro-inflammatory cytokines (214). The exo-miR-146a secreted by the DCs regulated the inflammatory response by inhibiting the IRAK-1 (215). Further, the CDCs-exo-miR-181b and BMMSCs-exo-miR-182 promoted the polarization of the M2 macrophages and thereby alleviated the inflammatory response (216, 217). The BMMSC-exo-miR-25, -miR-185, -miR-125b, and ADSCs-exo- miR-126 were also found to inhibit the inflammatory cytokines expression (207). Moreover, the exo-miR-223 and miR-210 could induce the expression of ICAM-1 to inhibit the inflammatory reaction (25, 32, 218).

The immune response participates in the pathogenesis of a variety of cardiovascular diseases, including AF. Anti-inflammatory has been validated maybe useful for the treatment of AF. EXo-ncRNA, as a new strategy for anti-inflammatory, should have important significance in the treatment of AF, but more researcsh are still needed.

#### Angiogenesis

The ADSCs-exo-miR-126 was found to promote the generation of microvascular cells and the migration of endothelial progenitor cells, through enhancing the VEGF pathway via the suppression of angiogenesis inhibitors SPRED1 and PI3KR2 (192). The EMSCs-exo-miR-21 could induce angiogenesis and improve the cardiac cells' survival via inhibiting the PTEN/Akt pathway (197). The BMSCs-exo-miR-210 and miR-132 could promote angiogenesis, inducing capillary-like tube formation and enhancing the migration and proliferation of HUVEC, through suppressing the expression of the Efna3 and *RASA1* (219–221). Moreover, several MSCs-exo-miRNAs including miR-30b, miR-30c, miR-424, and let-7 were identified to exert pro-angiogenic properties (178).

Promoting angiogenesis in ischemic areas is one of the important methods to improve MI. As previously stated, AAI can increase the susceptibility to AF, so promoting angiogenesis may be an important method for the treatment and prevention of AF. The exosomes-ncRNA may have an irreplaceable role in promoting angiogenesis.

## Exosome Enginnering for AF Treatment

### Direct Exosome Engineering

In direct encapsulation of cargoes into exosomes by sucrose gradient ultracentrifugation, Sun et al. used sucrose gradient ultracentrifugation successfully to encapsulate curcumin (a hydrophobic reagent) into the EL-4 cells-derived exosomes (222). However, this protocol can only be used for hydrophobic drugs. In order to address this, more active encapsulation techniques were applied, such as loading of catalase along with (1) incubation with and without saponin, (2) freeze-thaw cycles, (3) sonication and extrusion (223). Other processes like lipofection and electroporation have limited transfer efficiency and exosome concentration. As an alternative approach, the EVs-imitating structures were developed (173). Liposomes may be the most promising EV-imitating structure (224). Exosome delivery approaches mainly include intravenous injection or direct injection into the target area. Study found that injection of the liposomes into the infarct zone had significant anti-inflammatory, anti-fibrotic, and pro-angiogenetic effects (223).

### Indirect Exosome Engineering

Insufficient retainment in the myocardium is one of the major challenges in using exosomes for clinical applications. Currently, technologies for increasing exosomes retainment are being developed. Many targeting molecules have been developed for the exosome conjugation to enhance the retention and achieve the target delivery to the cardiac tissue. For example, Alvarez-Erviti et al. fused cardiomyocyte-specific binding peptide to the exosomal N-terminus of murine transmembrane protein Lamp2b to improve the cardiac tropism of the exosomes (225). Vandergriff et al. designed the myocardium-targeting exosomes with cardiac homing peptide (CHP) and found increased cells viability and exosomal uptake in the cardiomyocytes (226). The other example of indirect engineering is the manipulation of the loading mechanism to selectively load cargoes into the exosomes. Moreover, an attractive tool for protein delivery by the exosomes, which was based on the integration of a reversible protein interaction module was sensitive to blue light and led to the protein loading into exosomes (227). In addition, through transferring encoding genes to the parent cells, exosomes with enhanced production efficiency, specific packaging ability, and the delivery to target cells were developed, which comprised of a production booster, an active packaging device, and a cytosolic delivery helper (166, 228). The latest advances in biomaterials such as heart patches and hydrogels have made them the new favorites for endogenous repair treatments. Liu et al. loaded the exo-miRNAs into hydrogels and exploited them *in situ* to the rat hearts. This approach made the more sustainable exosomes with higher bioavailability, improved cardiac functions, and decreased CMs apoptosis (229). Studies by Vunjak-Novakovic et al. and Chen et al. reported similar results (166). Moreover, encapsulating the exosomes with the antioxidant peptides could enhance exosome targeting effects. Nevertheless, the targeted exosome delivery approaches with enhanced retention still need to be further explored. Moreover, those delivery approaches can be incorporated with a minimally invasive surgical approach such as CT or ultrasound guide tube pericardiostomy to reduce the risk associated with the treatment (Table 4).

**Table 4:** Current exosomes engineering techniques for Af treatments.

**Exosomes engineering technologies**	**Pros**	**Cons**
Encapsulate cargoes by sucrose gradient ultracentrifugation	Protect drugs from degradation, enhance drugs stability, bioavailability and effect	This protocol can only be used for hydrophobic drugs
Encapsulation cargoes through incubation, freeze-thaw cycles, sonication, and extrusion	Allows loading of both hydrophilic and hydrophobic drugs	Causes exosomal bilayer disruption
EV-imitating structure (liposomes)	Targeting, stable structure and contents	Physiochemical instability Can form unwanted degradants
Fusing cardiomyocyte-specific binding peptide to the exosomes (Cardiac homing peptide)	Enhance exosomes targeting	Displays only protein loading
Manipulation of the loading mechanism to selectively load cargoes into the exosomes (protein loading in exosomes based on integration of light sensitive reversible proteins interaction module)	Enhance exosomes targeting Controllable mechanism of loading	Displays only protein loading
Transfection of a gene encoding exosome-targeting proteins into parent cells.	Enhance production efficiency, specific packaging, and delivery to target cells	Displays only protein loading
Heart patches and hydrogels	Making exosomes release more sustained with higher bioavailability; enhance exosomes effects with better target	The delivery approaches with enhanced retention is unsatisfactory

Overall, exosomes prepared by exosome engineering may have a wide spectrum of prospects for the treatment of diseases including AF.

## Advantages and Disadvantages of Exosome for AF

Since the discovery of exosomes, studies on cardiovascular diseases (CVDs) have attracted extensive attention. In this review, we focused on the potential application of exosomes as diagnostic/prognostic and therapeutic tools in AF. Subsequently, we discussed the pros and cons of the use of exosomes. The application of exosomes has many advantages (32, 193, 230): (1) Alterations in exo-cargoes profile secreted by cardiac cells during AF would reflect the parental cells pathophysiological state with extreme specificity and sensitivity, and therefore they may appear as “fingerprint” of the AF pathogenetic processes; (2) Exosomes can be isolated from nearly all obtainable biofluids such as blood and urine; (3) Exosomes serve as a vehicle that protects cargoes from degradation and targets the cargoes to the recipient cells, with the less traumatic and abnormal modifications. (4) Well-designed engineered exosomes may enhance their therapeutic effects, making them promising tools for clinical application. (5) Exosomes therapy has fewer ethical issues, compared with stem cell therapy. Although the exosomes application for the AF has significant benefits, it also has some limitations (31, 202, 228): (1) Exo-RNAs in the circulating come from different tissues, so the source of exosomes cannot be completely determined, which may affect the specificity of the biomarkers for diagnosing AF. (2) The extraction and purification of exosomes are very complicated without a gold standard, and the efficiency is limited, moreover, the specificity and contents of exosomes are unstable. (3) The safety and toxicity of exosomes cannot be fully established. Although lower immunogenicity was reported, some cases may suffer fever or allergic and hemolytic reactions ect. (4) The delivery methods of the exosomes to the heart are sub-optimal. Moreover, even many techniques have been applied to improve the exosome targeting, but there is still the possibility of “off-target,” which may not only reduce efficiency but also cause additional side effects. (5) The dosage regimen of exosomes is not clear, and there are limitations on their pharmacokinetic parameters. (6) The exact exosomes' therapeutic effect is unclear, and how exosomes fulfill their specificity is yet to be fully understood.

Nowadays, exosomes have been extensively investigated in several pathological contexts such as ACS, MI, and HF diseases, but barely in the AF. However, as mentioned previously, as diagnostic biomarkers or treatment for AF, exosomes have many potential benefits, even if there are some limitations. Therefore, we need more elucidations to further clarify the exosomes' clinical value and side effects.

## Conclusion

In the past decade, research on exosomes biology, pathophysiological function, and potential clinical application has increased exponentially and provided novel knowledge in mechanisms and cargoes of exosomes, thereby providing an opportunity to use in the AF diagnosis and treatment. The review of preclinical and clinical studies concluded that the circulating exosomes containing cardiac-specific cargoes, especially ncRNAs, have great potential for the AF diagnosis/prognosis. Further, exo-ncRNAs have important therapeutic effects on AF pathogenesis. Exosome engineering can improve the distribution and selectivity to control the exosomal cargoes. Encapsulation technology has generated a platform for the effective delivery of synthetic and biopharmaceuticals. Therefore, the application of the exo-ncRNAs in the AF may have a good prospect. However, the exo-ncRNAs research related to the AF is still in its infancy, and many aspects need to be improved: (1) The isolation, characterization, and identification should be standardized and simplified. (2) Nomenclature should be consistent. (3) Exosomes should be quantified. (4) Further elaboration on the exosomes mechanism, improvement of targeting, reducing degradation, increasing retention needs to be elucidated in future research.

In conclusion, this review summarized the current biogenesis, isolation, biological functions, and future applications of the exosomes relevant to AF. Exosomes hold unprecedented opportunities for future applications for the AF either as biomarkers for diagnosis/prognosis or as therapeutic tools. Simultaneously, the challenges in the exosomes' application are also significant. Therefore, more prospective, large-scale, and multi-centered trials are needed before the exosomes can be used clinically in the AF. Undoubtedly, exosome-based application will herald a new chapter in clinical diagnosis/prognosis and treatment of AF.

## Author Contributions

CC and WZ researched the article and wrote the manuscript. QC, TZ, YP, YL, and YX reviewed and edited the manuscript before submission. All authors provided substantial contribution to the discussion of content.

## Funding

This work was supported by Shanghai Science and Technology Commission, Grant No. 17DZ1930303.

## Conflict of Interest

The authors declare that the research was conducted in the absence of any commercial or financial relationships that could be construed as a potential conflict of interest.

## Publisher's Note

All claims expressed in this article are solely those of the authors and do not necessarily represent those of their affiliated organizations, or those of the publisher, the editors and the reviewers. Any product that may be evaluated in this article, or claim that may be made by its manufacturer, is not guaranteed or endorsed by the publisher.
